# Sodium‐Glucose Cotransporter 2 Inhibitors for Metabolic Dysfunction‐Associated Steatohepatitis

**DOI:** 10.1111/1753-0407.70206

**Published:** 2026-03-06

**Authors:** Jiayang Lin, Yan Huang, Bingyan Xu, Chensihan Huang, Fei Teng, Youwen Yuan, Jinhua Zhang, Huijie Zhang

**Affiliations:** ^1^ Department of Endocrinology and Metabolism Nanfang Hospital, Southern Medical University Guangzhou China; ^2^ School of Public Health, Southern Medical University Guangzhou China; ^3^ Key Laboratory of Functional and Clinical Translational Medicine, Department of General Medicine Xiamen Medical College Xiamen China; ^4^ Department of Endocrinology and Metabolism Zhongshan Hospital, Fudan University Shanghai China

## Abstract

MASH denotes metabolic dysfunction‐associated steatohepatitis. MASH improvement was defined as a decrease in the non‐alcoholic fatty liver disease activity score (NAS) of ≥ 2 points or NAS ≤ 3 at week 48 after treatment, with no worsening of fibrosis (i.e., no increase in fibrosis stage). MASH resolution was defined as a hepatocellular ballooning score of 0 and a lobular inflammation score of 0 or 1 at week 48, with no worsening of fibrosis. Fibrosis improvement was defined as a reduction in fibrosis of ≥ 1 stage at week 48, with no worsening of MASH (i.e., no increase in steatosis, ballooning, or inflammation scores).

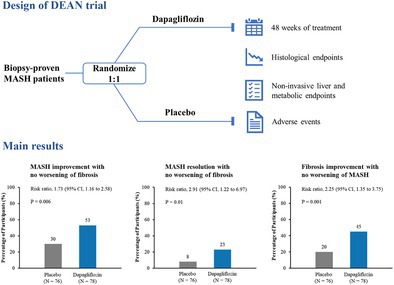

Metabolic dysfunction‐associated steatohepatitis (MASH) is a severe and progressive subtype of metabolic dysfunction‐associated steatotic liver disease (MASLD) [1]. Closely associated with insulin resistance, MASH affects more than 30% of the patients with type 2 diabetes (T2D) and may progress to cirrhosis in up to 25% of cases [2, 3]. Despite decades of research since the 1980s, hundreds of drug development programs have failed, and to date, only two agents (thyroid hormone receptor‐beta agonist resmetirom and glucagon‐like peptide‐1 receptor agonist semaglutide) have received U.S. approval for MASH [4, 5]. Given this unmet need, drug repurposing has emerged as a promising strategy, particularly for agents already in clinical use. Regarding the strong pathophysiological overlap between MASH and T2D, anti‐diabetic drugs are considered to have potential as anti‐MASH drugs. Sodium‐glucose cotransporter 2 (SGLT2) inhibitors have consistently shown benefits in MASLD, as evidenced by improvements in non‐invasive liver parameters [6]. Mechanistically, these agents result in weight loss by increasing urinary glucose secretion and appear to induce a fasting‐like metabolic paradigm and modulate nutrient‐sensing pathways [7]. In the liver, SGLT2 inhibitors promote the synthesis of ketone bodies, which in turn reduces hepatic infiltration and impairs the effector functions of CD8^+^ T cells, thereby ameliorating the progression of MASH [8]. A plausible mechanistic framework for these effects involves the systemic metabolic reprogramming and negative energy balance induced by SGLT2 inhibition, along with potential direct actions on MASH pathogenesis [7]. Nevertheless, clinical evidence for their efficacy in MASH has been lacking, largely because confirmed diagnosis of MASH requires liver biopsy. This gap was recently addressed by the first RCT demonstrating that the SGLT2 inhibitor dapagliflozin improves histological outcomes in patients with MASH and fibrosis [9].

This multicenter, double‐blind, randomized, placebo‐controlled trial (DEAN trial) enrolled 154 adults (45% with T2D) with biopsy‐confirmed MASH. Participants received dapagliflozin 10 mg or placebo once daily for 48 weeks, with paired liver biopsy obtained at baseline and study end. The primary endpoint, MASH improvement (≥ 2 point NAS [non‐alcoholic fatty liver disease activity score] reduction or NAS ≤ 3) without fibrosis worsening, was achieved significantly more frequently with dapagliflozin (53%) than placebo (30%) (RR 1.73, 95% CI 1.16–2.58; *p* = 0.006). Dapagliflozin also demonstrated superiority in two key regulatory secondary endpoints: MASH resolution without fibrosis worsening (23% vs. 8%; RR 2.91, *p* = 0.01) and fibrosis improvement without MASH worsening (45% vs. 20%; RR 2.25, *p* = 0.001). Improvements were also observed in non‐invasive liver parameters and metabolic parameters. Mediation analysis indicated that weight loss largely mediated dapagliflozin's effects on MASH improvement and resolution, but not on fibrosis improvement, suggesting additional, possibly direct antifibrotic mechanisms of SGLT2 inhibitors. Adverse events were similar between the two groups. However, in this trial, a relatively high proportion of younger and male patients was enrolled, likely because they are more willing to undergo liver biopsy and often present with more advanced MASH, suggesting that the study population may represent patients with more active disease. Moreover, the findings should not be directly generalizable to non‐Asian populations, though current evidence does not indicate significant ethnic differences in MASH pathogenesis or treatment response. Nonetheless, this trial provides histological evidence that 48‐week treatment with dapagliflozin is superior to placebo for MASH management.

Despite limitations in generalizability and the absence of formal regulatory approval for MASH, SGLT2 inhibitors are already recommended as first‐line therapy for T2D [10]. This underscores dapagliflozin as an important therapeutic option for patients with comorbid T2D and MASH, alongside other glucose‐lowering agents (e.g., semaglutide) that have demonstrated histological improvements in MASH and fibrosis. Notably, a prespecified subgroup analysis of DEAN trial indicated that participants with T2D demonstrated a greater benefit in fibrosis improvement without MASH worsening compared to those without T2D [9]. Furthermore, population‐based cohort studies in patients with T2D have consistently associated SGLT2 inhibitor use with lower risks of liver‐related outcomes, including cirrhosis and hepatocellular carcinoma [11, 12, 13]. Together, these data extend the well‐established cardio‐renal‐metabolic protective profile of SGLT2 inhibitors to include meaningful hepatic protection in patients with T2D [14].

In recent years, the therapeutic landscape for MASH has expanded rapidly, encompassing THR‐β agonists [4], incretin‐based agents (GLP‐1 receptor agonists [5], dual/triple agonists [15, 16]), SGLT2 inhibitors [9], and emerging compounds such as pan‐PPAR agonists [17] and FGF21 analogs [18, 19]. Although none have yet demonstrated conclusive benefits on clinical liver outcomes, these agents demonstrate promising evidence for improving histological outcomes of MASH and liver fibrosis. Among them, SGLT2 inhibitors hold a unique distinction based on their well‐documented cardio‐renal‐metabolic benefits, oral administration, and cost‐effectiveness—advantages particularly relevant in patients with T2D. While direct head‐to‐head trials are lacking, indirect comparisons suggest that dapagliflozin yields a moderate effect on MASH resolution without fibrosis worsening (net placebo‐adjusted difference: 15%), compared with 29% for semaglutide and 20% for resmetirom. Conversely, dapagliflozin shows a more pronounced effect on fibrosis improvement without MASH worsening (25%), compared with 14% for semaglutide and 12% for resmetirom [4, 5]. These findings must be interpreted cautiously with the absence of comparative data. Nevertheless, confirmation of the efficacy of dapagliflozin on clinical liver outcomes (e.g., cirrhosis or hepatocellular carcinoma) in MASH patients awaits large‐scale and long‐term randomized controlled trials. Additionally, it remains unclear whether the beneficial effects of dapagliflozin on MASH represent a class effect of SGLT2 inhibitor, whereas biomarker‐based evidence in MASLD showed consistent benefits of other SGLT2 inhibitors (e.g., empagliflozin, canagliflozin, and ipragliflozin) [20, 21].

Beyond pharmacological innovation, future research directions are likely to place greater emphasis on personalized medicine, combination therapies, comorbidity management, and prevention of MASH and fibrosis progression. Critically, sustained lifestyle intervention remains the cornerstone of MASH management. Without successful lifestyle modifications, MASH patients may necessitate long‐term, potentially lifelong pharmacological treatment to maintain benefit.

## Author Contributions

J.L., and H.Z., conceptualized and wrote the manuscript. Y.H., B.X., C.H., F.T., Y.Y., and J.Z. reviewed and approved the final versions of the manuscript. H.Z. is the guarantor of this work.

## Funding

This work was supported by Noncommunicable Chronic Diseases–National Science and Technology Major Project (2023ZD0508300). National Science Fund for Distinguished Young Scholars (82325011). Guangzhou Science and Technology Plan Project (SL2024B03J00202). Xiamen Municipal Medical and Health Science and Technology Program (3502Z20224ZD1296). Fujian Provincial Natural Science Foundation of China (2023J01130388). National Natural Science Foundation of China (82470911). Joint Funds of the National Natural Science Foundation of China (U22A20288).

## Conflicts of Interest

The authors declare no conflicts of interest.

## Data Availability

Data sharing not applicable to this article as no datasets were generated or analysed during the current study.
